# Enhancement of Yield, Phytochemical Content and Biological Activity of a Leafy Vegetable (*Beta vulgaris* L. var. *cycla*) by Using Organic Amendments as an Alternative to Chemical Fertilizer

**DOI:** 10.3390/plants12030569

**Published:** 2023-01-27

**Authors:** Angela Libutti, Daniela Russo, Ludovica Lela, Maria Ponticelli, Luigi Milella, Anna Rita Rivelli

**Affiliations:** 1 Department of Science of Agriculture, Food, Natural Resources and Engineering, University of Foggia, Via Napoli, 25, 71122 Foggia, Italy; 2 Department of Sciences, University of Basilicata, Via dell’Ateneo Lucano, 10, 85100 Potenza, Italy; 3 Spinoff BioActiPlant s.r.l., Via dell’Ateneo Lucano, 10, 85100 Potenza, Italy; 4 School of Agricultural, Forest, Food and Environmental Sciences, University of Basilicata, Via dell’Ateneo Lucano, 10, 85100 Potenza, Italy

**Keywords:** Swiss chard, biochar, vermicompost, yield, nitrate, polyphenols, health-promoting compounds, antioxidant activity

## Abstract

This study evaluates the effect of a chemical fertilizer (ammonium nitrate), a compost (vermicompost from cattle manure) and two biochars (from vine prunings and wood chips, respectively), applied to the soil alone or in mixture, on the yield, phytochemical content and biological activity of *Beta vulgaris* L. var. *cycla* (Swiss chard). The respective treatments, each replicated four times, were arranged according to a completely randomized block design. Results showed that vermicompost, both alone and in mixture with vine pruning biochar, significantly increased yield parameters (plant height and leaf area) and yield over the untreated soil and the biochars alone, similar to ammonium nitrate. Moreover, vermicompost, both alone and in mixture, respectively, with the two biochars, determined lower total N and NO_3_^−^ contents than ammonium nitrate, both alone and in mixture, respectively, with the two biochars. In particular, NO_3_^−^ content was within the safe thresholds fixed for leafy vegetables by the European Commission to prevent any adverse implication on human health from dietary NO_3_^−^ exposure. The biochars alone resulted in very low yield and leaf total N content, likely due to a limited release of N for plant uptake, also evidenced by the undetectable NO_3_^−^ leaf content, similarly shown by plants grown in untreated soil. Vermicompost, alone or in mixture, respectively, with the two biochars, increased the content of specialized metabolites, with a positive effect on antioxidant activity. The organic amendments, particularly compost, could be an alternative to chemical fertilizers to reach a trade-off between yield, nutritional and health qualities in Swiss chard, meeting the needs of farmers and consumers as well as the targets for sustainable food production.

## 1. Introduction

Leafy vegetables constitute a wide variety of species of great importance in the daily human diet due to their high nutritional content, including minerals, proteins and vitamins [1]. They are also beneficial for human health and wellness because of their content of multitudinous phytochemicals, such as antioxidants, polyphenols and other non-nutritive bioactive metabolites, which help to lower the risks of the occurrence of certain chronic diseases [2]. In this regard, the role of the regular consumption of vegetables, particularly green leafy vegetables, in preventing or delaying the onset of cancer, type 2 diabetes and cardiovascular diseases late in life has now been accepted and confirmed by epidemiological studies [3]. 

Among the leafy vegetables is *Beta vulgaris* L. var. *cycla*, a species belonging to the Amaranthaceae family, native to the coasts of the Mediterranean basin, also known by the common name Swiss chard. The Amaranthaceae family also includes spinach (*Spinacia oleracea* L.), the most important and consumed leafy vegetable in Europe. Swiss chard has been used as plant-based food since 1000 B.C. by the Mediterranean population, particularly the Romans. Today, worldwide, its edible leaves and stalks are eaten raw in salads or cooked. Depending on the genotype, the leaves may be smooth or curly, with white, yellow, orange or red stalks. The cultivation of this species is widespread in the USA, South America, northern India and the Mediterranean countries, particularly south Italy. Production reaches its peak in early summer and fall, although the leaves are available at markets year-round, thanks to greenhouse cultivation. Particularly in summer, the leaves are consumed in place of other leafy species not easily available in this season, such as spinach. Swiss chard is easier to grow than spinach and has the characteristics of rapid growth and high yield. It is very suitable for cultivation as a forecrop and an aftercrop in crop rotation. The content of nutrients and phytochemicals makes this species very attractive as a beneficial plant-based food [4]. Both leaves and stalks contain a high amount of minerals (Ca, Mg, K, Mn and Fe) and vitamins (A, B, C) [5]; they also provide a good supply of proteins and dietary fibers that contributes to the role in sugar blood regulation [5,6]. Moreover, Swiss chard is rich in chlorophyll and carotenoids, which represent very beneficial phytopigments in improving the antioxidant, detoxification and immune defense systems, once assumed from the diet [7]. It also contains considerable concentrations of compounds with high free-radical scavenging and antioxidant activity, such as phenolic acids (caffeic, p-cumaric, and syringic) and flavonoids (apigenin-derived glycosides, kaempferol and quercetin from apigenin) [5,6,8]. Present in Swiss chard are also betalains, identified for the first time in differently colored varieties by Kugler et al. [9]. Betalains, the main source of which is beetroot (*Beta vulgaris* L.), are biologically active compounds with an important health role, deriving from their high antioxidant scavenging and anticancer and chemo-preventive potential [10]. 

The nutrient and phytochemical contents of leafy vegetables are highly variable among the different species, and this diversity is of high significance for ensuring food and nutritional security [11]. The biosynthesis of such compounds is affected by genotype, environmental and growing conditions, agricultural management practices and harvest and post-harvest conditions [12,13,14,15,16,17]. Fertilization, particularly nitrogen rate and form, is of crucial importance in driving the plant biosynthesis of biologically active compounds [18,19,20]. Additionally, nitrogen is a major essential macronutrient for plant growth and product quality in leafy vegetables [21,22]. These species are considered very nitrogen-demanding due to the large amounts of this nutrient they require in a short period of time. Nitrogen is easily taken by plants even in excessive amounts, and its concentration in plant tissues increases as nitrogen content in the substrate increases. In this regard, Swiss chard is reported as a high nitrate accumulator compared to other vegetables (root and fruit vegetables). Together with lettuce and spinach, it represents a major source of dietary nitrate intake [23], 5–7% of which is reduced to nitrites by oral and gastric bacteria in adults; in infants, the conversion rate is even higher. Nitrite and other metabolites and reaction products of nitrate, such as NO and N-nitrosamine, are of great health concern, representing risk factors for the onset of blue-baby syndrome (or methemoglobinemia) and cancer [24]. To prevent this human health hazard, the WHO (World Health Organization) recommends a 3.7 mg NO_3_^−^/kg body weight daily intake. A number of studies [25,26,27,28] have shown that the application of high rates of chemical nitrogen fertilizers to Swiss chard, though increasing plant yield, negatively affects the quality of the fresh product. Proper nitrogen fertilization might have a trade-off effect between yield and qualitative traits (particularly nitrate accumulation) and the concentration of phytochemicals in leafy vegetables. 

According to the “Farm to Form Strategy” [29], proposed by the European Commission as a part of the Green Deal [30], a reduction by at least 20% of fertilizer application and an increase by at least 25% of organically farmed land should be achieved by 2030 in the European Union countries. Within such a context, the use of organic amendments in place of chemical N fertilizers could meet the needs of farmers, consumers and the targets for sustainable food production [31,32,33,34,35]. Among the organic amendments, compost that derives from a controlled process of aerobic decomposition and the stabilization of different kinds of organic wastes (plant material or manure) [34] and biochar that derives from a pyrolysis process under partial or total anaerobic conditions of different types of organic biomasses (crop residues, wood biomasses, animal wastes, sewage sludges) [35,36,37,38,39] are of significant interest, as documented by the growing number of studies published in scientific journals included in the Web of Science database in the last decades (2007–2022) [40]. 

Focusing on Swiss chard, several studies have reported the positive, negative or neutral effects of different organic amendment types and doses on the quantitative–qualitative response of this crop [41,42,43,44]. In previous experiments, where soil was treated with biochar and different compost types, used alone [45] or in mixture [46], we found that vermicompost and compost, respectively deriving from cattle manure and cattle anaerobic digestate, improved the growth of Swiss chard compared to compost deriving from olive pomace and biochar obtained from vine pruning residues. Furthermore, a following experiment [47], which evaluated the co-application of a biochar from wood chips and a biochar from vine prunings, respectively, with vermicompost from cattle manure (as organic fertilizer) or ammonium nitrate (as chemical fertilizer), showed again the significant effect of vermicompost on both the number and fresh weight of leaves in all five harvests performed during the growth cycle; this effect was similar to that of ammonium nitrate. Moreover, vermicompost limited leaf NO_3_^−^ accumulation compared to ammonium nitrate, while the two biochars increased the leaf content of PO_4_^3−^, SO_4_^2−^, Na^+^, Ca^2+^, K^+^ and Mg^2+^, the major beneficial nutrients for human health. 

However, very limited is the available literature about the phytochemical content and biological activity of Swiss chard cultivated using compost and biochar [5,6,8,48]. Following our previous findings regarding the influence of these organic amendments on leaf production as well as the leaf nutrient and NO_3_^−^ contents of this species, we hypothesized that they could also affect the biosynthesis of biologically active compounds. If a positive effect should be detected, their use in the production of Swiss chard could represent a very effective alternative to chemical fertilizers in order to reach a profitable yield while obtaining a vegetable product with appreciable nutritional and health values. 

Therefore, the present study is aimed at comparing the effect of a chemical fertilizer (ammonium nitrate), a compost (vermicompost from cattle manure) and two biochar types (from vine prunings and wood chips, respectively), applied to the soil alone or in mixture (biochar–ammonium nitrate and biochar–vermicompost), on the total yield, phytochemical content and biological activity of Swiss chard.

## 2. Results

### 2.1. Yield, Water Consumption and Water-Use Efficiency 

Among the considered yield parameters (Table 1), plant height (H) and leaf area (LA) were significantly affected by the experimental treatments. More specifically, the application of ammonium nitrate and vermicompost, both alone (F and V, respectively) and in a mixture with biochar from vine prunings (Bv+F and Bv+V, respectively), resulted in significantly (*p* ≤ 0.001) higher H and LA values over the untreated soil or control (C) and the two biochars applied alone (Bw and Bv, respectively). In contrast, the application of ammonium nitrate and vermicompost in the mixture with biochar from wood chips (Bw+F and Bw+V, respectively) resulted in H and LA values not statistically different from C, Bw and Bv. The leaf dry matter content (DM) did not show any significant difference among the considered experimental treatments, varying from 6.9% to 8.1%. Additionally, considering the yield (Table 1), a significant and similar effect (*p* ≤ 0.001) of F, V, Bv+F and Bv+V treatments in enhancing the productivity of Swiss chard was clearly detected, as compared to C, Bw and Bv, which did not differ from each other. Very interestingly, considering the average yield of F, V, Bv+F and Bv+V treatments, an increase of 140% in comparison to the average yield of C, Bw and Bv treatments was detected (2638 vs. 1102 g m^−2^). The yield increase corresponded to water consumption (Wc) over the growth cycle (Table 1) equal to 165 L m^−2^, as the average of the F, V, Bv+F and Bv+V treatments, which was only 1.2 times higher than the average Wc detected in C, Bw and Bv (143 L m^−2^), and to water-use efficiency (WUE) (Table 1) that was 2.1 times higher in the F, V, Bv+F and Bv+V treatments (on average, 16 g fw L^−1^) than C, Bw and Bv (on average, 7.7 g fw L^−1^). 

Considering the Wc and WUE values of each experimental treatment, as reported in Table 1, both Bw- and Bv-treated plants showed lower water consumption, although statistically different (*p* ≤ 0.01) only from Bv+F, while the WUE followed the trend already observed for H, LA and yield, showing significantly (*p* ≤ 0.001) higher values in F-, V-, Bv+F- and Bv+V-treated plants than C, Bw and Bv.

### 2.2. SPAD Index, Total N and NO_3_^−^ Content of Leaves 

**Figure 1:**
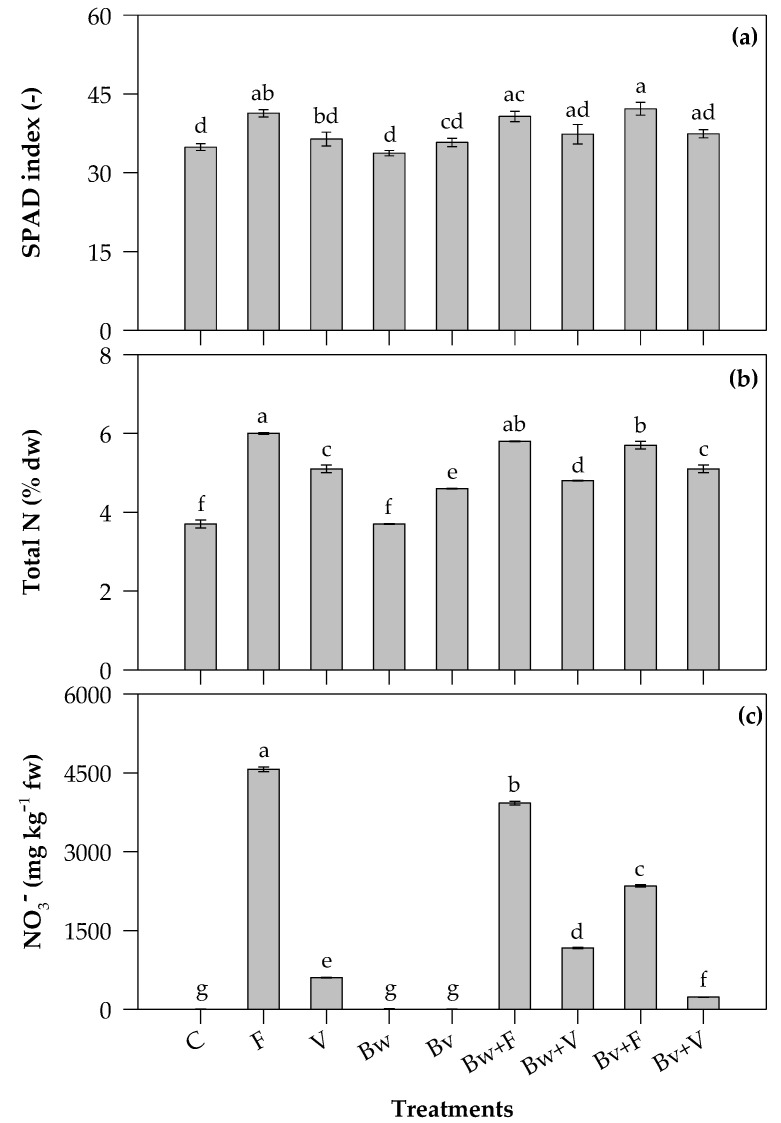
SPAD index (**a**), total nitrogen (total N, % dw) (**b**) and nitrate (NO_3_^−^, mg kg^−1^ fw) (**c**) contents of Swiss chard leaves. C, control; F, ammonium nitrate; V, vermicompost; Bw, biochar from wood chips; Bv, biochar from vine prunings; Bw+F and Bv+F, biochars respectively mixed with ammonium nitrate; Bw+V and Bv+V, biochars respectively mixed with vermicompost. Values are means (n = 4) ± s.e. Different letters above histograms indicate significant differences among treatments (*p* ≤ 0.05; Tukey’s test).

The SPAD index significantly (*p* ≤ 0.001) differed among the experimental treatments (Figure 1a). More specifically, a higher SPAD index was observed in both F- and Bv+F-treated plants (on average, 42) than C, Bw and Bv, which did not differ from each other (on average, 35), accounting for an increase of 20%. The SPAD index did not differ between F and V, showing, on the contrary, a 16% higher value in Bv+F (42) than V (36). Moreover, a higher SPAD index was observed in Bw+F-treated plants (41) than C and Bw, which did not differ from each other (on average, 34), accounting for a 19% increase.

Additionally, the total N leaf content was significantly (*p* ≤ 0.001) influenced by the experimental treatments (Figure 1b). As expected, plants treated with F, as well as Bw+F and Bv+F, resulted in higher leaf total N content, followed by plants treated with V, the two mixtures Bw+V and Bv+V, the two biochars Bw and Bv and the untreated plants. Compared to C, plants treated with F, Bw+F and Bv+F showed 62%, 57% and 54% leaf total N increases, respectively, versus 38%, 31% and 39% lower increases in V-, Bw+V- and Bv+V-treated plants, respectively. 

The significant (*p* ≤ 0.001) influence of the experimental treatments was also detected when considering the leaf NO_3_^−^ content (Figure 1c). Expectedly, the NO_3_^−^ reached the highest content in plants treated with F, followed by the two mixtures Bw+F and Bv+F. In contrast, plants treated with V resulted in a very lower NO_3_^−^ leaf content, accounting for an 87% NO_3_^−^ decrease in comparison to F (603 vs. 4568 mg kg^−1^ fw). When plants were treated with the mixture Bv+V, leaf NO_3_^−^ content further decreased, showing the lowest value (231 mg kg^−1^ fw) and accounting for a 95% decrease in comparison to F. Instead, plants treated with the mixture Bw+V showed a higher leaf NO_3_^−^ content than V and Bv+V, accounting for a 74% decrease in comparison to F (1166 vs. 4568 mg kg^−1^ fw). Moreover, both Bv+V and Bw+V treatments resulted in about 3 and 10 times lower leaf NO_3_^−^ contents than Bw+F and Bv+F, respectively. No differences among C, Bw and Bv treatments were observed due to a very low NO_3_^−^ content in leaves, the amount of which was below the instrumental detection limit.

### 2.3. Phytochemical Contents of Leaves

**Figure 2:**
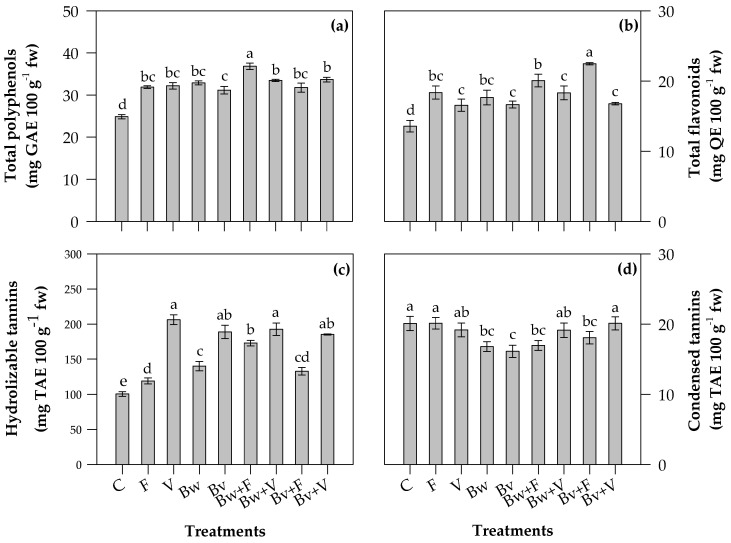
Total polyphenol (mg GAE 100 g^−1^ fw) (**a**), total flavonoid (mg QE 100 g^−1^ fw) (**b**), hydrolyzable tannin (mg TAE 100 g^−1^ fw) (**c**) and condensed tannin (mg TAE 100 g^−1^ fw) (**d**) contents of Swiss chard leaves. C, control; F, ammonium nitrate; V, vermicompost; Bw, biochar from wood chips; Bv, biochar from vine prunings; Bw+F and Bv+F, biochars respectively mixed with ammonium nitrate; Bw+V and Bv+V, biochars respectively mixed with vermicompost. Values are means (n = 3) ± s.e. Different letters above histograms indicate significant differences among treatments (*p* ≤ 0.05; Tukey’s test).

The content of polyphenols, flavonoids and hydrolyzable tannins in the leaves of all the tested treatments was found to be higher than C, showing that the organic amendments and chemical fertilizer increased the amount of specialized metabolites (Figure 2). 

In particular, the highest content of polyphenols (Figure 2a) was found in Bw+F-treated plants, with an amount of 36.8 ± 0.3 mg GAE 100 g^−1^ fw. The use of V, F and biochars, both alone and in mixture with F and V, showed no significant differences. The chemical fertilizer increased the content of total flavonoids (Figure 2b) in leaves compared to V. In fact, flavonoid content was found to be higher in Bv+F (22.5 ± 0.2 mg QE 100 g^−1^ fw) and Bw+F (20.1 ± 0.9 mg QE 100 g^−1^ fw) treatments, the results not being statistically different from the single components, F and Bw. 

Vermicompost alone (206.2 ± 6.96 mg TAE 100 g^−1^ fw) and in mixture with both the biochars significantly contributed to hydrolyzable tannins (Figure 2c) by increasing their content in leaves up to twice the amount compared to C (100.4 ± 3.3 mg TAE 100 g^−1^ fw) and chemical fertilizer (119.0 ± 4.3 mg TAE 100 g^−1^ fw). 

Regarding condensed tannins (Figure 2d), the F and V treatments reported similar or lower content than C, indicating that the Bw and Bv applications reduced the content of condensed tannins; no significant difference was observed in condensed tannin content among the other treatments. 

### 2.4. Antioxidant Activity 

**Figure 3:**
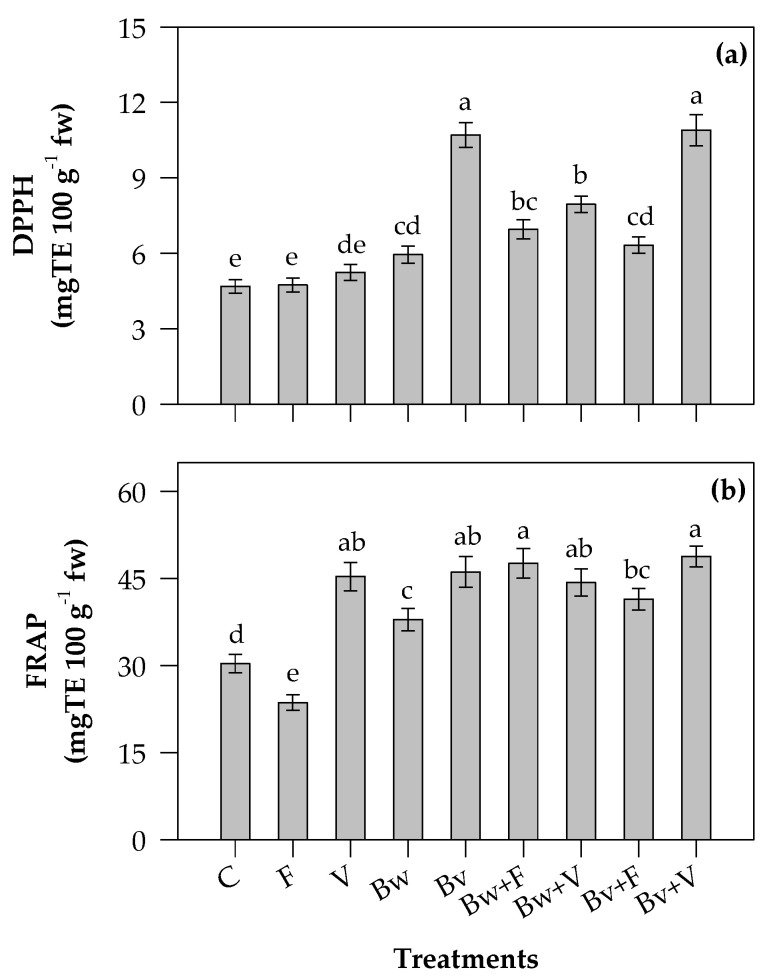
Antioxidant activity tested by the 2,2-diphenyl-1-picrylhydrazyl content (DPPH, mg TE 100 g^−1^ fw) (**a**) and reducing power (FRAP, mg TE 100 g^−1^ fw) (**b**) of Swiss chard leaves. C, control; F, ammonium nitrate; V, vermicompost; Bw, biochar from wood chips; Bv, biochar from vine prunings; Bw+F and Bv+F, biochars respectively mixed with ammonium nitrate; Bw+V and Bv+V, biochars respectively mixed with vermicompost. Values are means (n = 4) ± s.e. Different letters above histograms indicate significant differences among treatments (*p* ≤ 0.05; Tukey’s test).

The specialized metabolites exhibited important beneficial effects, including antioxidant activity involved in the reduction of free radicals, which are responsible for several diseases, or decreasing their rate of production,. Antioxidant activity was evaluated using two different assays, measuring radical-scavenging activity (DPPH) and reducing power (FRAP) (Figure 3).

The DPPH-scavenging activity (Figure 3a) of leaves ranged from 4.7 ± 0.3 (C) to 10.9 ± 0.6 mg TE 100 g^−1^ fw (Bv+V). Vermicompost alone (5.2 ± 0.3 mg TE 100 g^−1^ fw) was comparable to F (4.7 ± 0.3 mg TE 100 g^−1^ fw) and C (4.7 ± 0.3 mg TE 100 g^−1^ fw). The values obtained from plants treated with the combinations Bw+F (6.95 ± 0.37 mg TE 100 g^−1^ fw), Bv+F (6.32 ± 0.32 mg TE 100 g^−1^ fw) and Bw+V (7.95 ± 0.32 mg TE 100 g^−1^ fw) were not significantly different.

The reducing power (Figure 3b) ranged from 23.6 ± 1.3 mg TE (F) to 48.8 ± 1.8 mg TE 100 g^−1^ fw (Bv+V). The use of vermicompost (V) significantly increased the reducing power, both alone and in mixture with the biochars. The chemical fertilizer negatively affected antioxidant activity by FRAP assay. 

**Figure 4:**
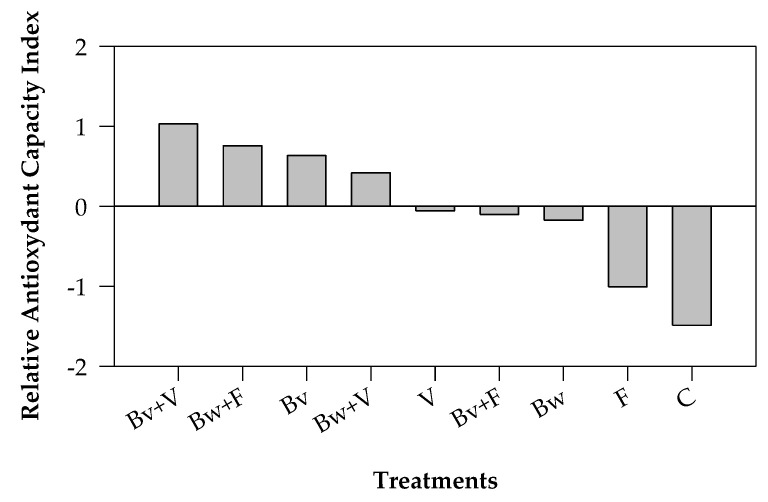
Relative Antioxidant Capacity Index (RACI). C, control; F, ammonium nitrate; V, vermicompost; Bw, biochar from wood chips; Bv, biochar from vine prunings; Bw+F and Bv+F, biochars respectively mixed with ammonium nitrate; Bw+V and Bv+V, biochars respectively mixed with vermicompost.

The comparison of antioxidant assays, including TPC based on the redox reaction, allowed us to determine the Relative Antioxidant Capacity Index (Figure 4), an integrated approach to evaluating food antioxidant capacity. It is a relative value, not representing the specific antioxidant ability of food, but RACI has been found to be an accurate ranking of antioxidant capacity among foods. Considering all treatments, the vermicompost mixed with Bv provided the highest RACI, while the chemical fertilizer gave a lower value, close to untreated soil.

## 3. Discussion

### 3.1. Swiss Chard Yield

The application of ammonium nitrate and vermicompost from cattle manure positively affected the yield parameters and total yield of Swiss chard. Interestingly, both the chemical fertilizer and organic amendment resulted in a similar plant response, similarly increasing plant height, leaf area and yield. These findings agree with our previous results [47], where vermicompost was similarly effective to ammonium nitrate in determining a significant increase in the number and fresh weight of leaves per plant. They further confirm the beneficial effect of vermicompost on plant productive performance, highlighting the fertilizing value of this organic amendment [46]. According to Lazcano et al. [49] and Joshi et al. [50], vermicompost has a high nutritional value due to earthworm action that increases the mineralization rate and humification degree of feedstocks, which allows a richer end product than compost. This amendment is a rich source of plant nutrients in a readily available form (such as potassium, nitrogen, magnesium, phosphorus and calcium) and plant growth regulators; it also contains functional microorganisms, particularly bacteria, actinomycetes and fungi, and is characterized by a highly porous structure with high water-holding capacity and aeration. There are many studies reporting the effectiveness of vermicompost in increasing the productivity of many horticultural crops, including spinach [51], lettuce [52], tomato [53] and a number of other crops, such as cereals and legumes, fruit, aromatic, medicinal, ornamental and forestry plants [50]. Our results suggest that vermicompost can be used as a chemical fertilizer substitute in Swiss chard production for a more sustainable cultivation practice, allowing us to reduce the application of nitrogen fertilizers that are often the cause of environmental pollution [54]. In this regard, the findings of the present experiment also corroborate the results reported in previous studies on other cultivated plants [25,55,56]. 

Contrary to vermicompost, both the biochar from vine prunings and the biochar from wood chips, when applied alone, resulted in lower yield, likely due to low nutrient content and release capacity [57]. Consistent with our previous findings [45,46,47], these results suggest that, among organic amendments, biochar is not always a viable option to achieve sustainable crop production. Indeed, it is now well established that biochar’s impact on the growth and yield of crops is highly influenced by several factors, such as feedstock types, pyrolysis conditions, the physical structure and chemical composition of the biochar, properties of the cultivated soil [58],the interaction between the biochar and soil [59] and the tested crops [60], leading to highly variable [61] and not always consistent results [59,62]. 

However, our results show the positive effect of the vine pruning biochar and vermicompost mixture on plant productivity, resulting in similar plant heights, leaf areas and yields to those of ammonium nitrate and vermicompost due to the soil nutrient availability being improved by the nutrient-holding ability of biochar [31,33,63]. Particularly interesting is the positive effect of the biochar and vermicompost mixture because it highlights the advantage derived from putting together the ability of biochar to retain nutrients and the capacity of compost to release nutrients for plant uptake. In this regard, we supposed that the highly porous structure that usually characterizes biochar [64] likely enhanced the adsorption of the nutrients released by vermicompost, particularly reducing the losses of volatile and/or leachable nutrients (e.g., NH_4_^+^, NO_3_^−^), thus increasing their availability for the plants. The findings of our study agree with the results reported by Huang et al. [65] on basil and tomato plants. They found enhanced plant growth when the two species were grown in substrates obtained by mixing different rates of biochar (20%, 40%, 60% and 80%, by volume) with different rates of vermicompost (5%, 10%, 15% and 20%, by volume) compared to a commercial peat-based substrate. Additionally, Alvarez et al. [66] showed higher Petunia and Pelargonium growth and flower production in mixtures of biochar (8–12% by volume) and vermicompost (10–30% by volume) than the standard growing media.

Consistent with the enhanced Swiss chard yield was the improved water-use efficiency observed in plants treated with both ammonium nitrate and vermicompost, as well as in plants treated with the mixture of biochar from vine prunings and ammonium nitrate or vermicompost.

### 3.2. SPAD Index, Total N and NO_3_^−^ Content of Leaves 

The SPAD index can quickly and non-destructively carry out plant nitrogen nutrition diagnosis and predict the content of chlorophyll in leaves [67]. Indeed, it is closely related to the leaf content of nitrogen, which is the main component of chlorophyll molecules [68]. In our study, the SPAD index values were in accordance with those reported for Swiss chard and other leafy vegetables by other authors [14]. They also agreed with our previous findings [45]. The application of ammonium nitrate, both alone and in mixture with the two biochars, increased the SPAD index value due to enhanced nitrogen availability in the soil and increased plant nitrogen uptake, which improved the leaf chlorophyll content. On the contrary, the two biochars alone reduced the SPAD index value due to very low nitrogen availability to the plants, as showed by the similar SPAD index values registered in untreated plants. 

Still referring to our results, the total nitrogen content of leaves was greatly increased by the ammonium nitrate application, confirming that fertilization is a key factor driving the amount of this macronutrient in Swiss chard leaves [8]. The leaf nitrogen content reached higher values in all plants grown on soil treated with ammonium nitrate, both applied alone and in mixture with the two biochars, in accordance with SPAD index values. In contrast, the application of vermicompost and the corresponding mixtures with biochars lowered the total nitrogen content. This is an interesting result, further highlighting the vermicompost’s potential to act as a good substitute for chemical fertilizer in Swiss chard cultivation [47]. Indeed, the use of vermicompost has the beneficial effect of avoiding excessive nitrogen plant uptake while increasing plant yield to the same level as ammonium nitrate. The two types of biochar showed even lower leaf nitrogen contents, and this was probably due to the insufficient release of N for plant uptake. This led us to suppose the very limited fertilizing value of the two considered biochars, which did not serve as adequate N sources for plant development and production.

The effect of chemical fertilizers is also reflected in the leaf nitrate content. Plants treated with ammonium nitrate showed the highest NO_3_^−^ content, which was within the safety thresholds fixed by the European Commission (Regulation No. 1258/2011) in order to prevent potential adverse effects on human health from the consumption of leafy vegetables: rocket (6000–7000 mg kg^−1^ fw) and lettuce (3000–5000 mg kg^−1^ fw), although it exceeded the threshold set for spinach (2500–3500 mg kg^−1^ fw). Moreover, this nitrate content was higher than the NO_3_^−^ content reported for Swiss chard by the European Food Safety Authority (EFSA) [69] and a survey on leafy vegetables [70], indicating values ranging from 12 to 1078 mg NO_3_^−^ kg^−1^ fw. On the contrary, when plants were treated with the mixtures of the two biochars and ammonium nitrate, leaf nitrate content tended to be reduced, likely due to the occurrence of a sorption of nitrates on the biochar surface. Several authors have shown that biochar is effective in retaining nitrates in the soil [31,33,71,72] due to the high temperature (>600 °C) under which pyrolysis takes place [73], which gives a high surface charge area and micropore amount to the end product, thus enhancing its retention ability [73,74,75].

The leaf nitrate content further decreased following vermicompost soil application, both alone and in mixture with the two biochars, showing the high effectiveness of these organic amendments in also limiting nitrate accumulation in Swiss chard leaves. In this regard, the leaf nitrate content was well within the ranges reported by previous studies for organically cultivated Swiss chard [76,77].

### 3.3. Phytochemical Contents and Antioxidant Properties of Leaves

Polyphenols are one key class of carbon-based specialized metabolites with ecological and nutritive importance. Structurally, they are characterized by multiple phenolic hydroxyl groups, including flavonoids and tannins (hydrolyzable and condensed). Plants interact with the external environment through them. The biosynthetic pathways of polyphenols include the shikimic acid and phenylpropanoid metabolism pathways, with phenylalanine and tyrosine as precursor compounds. 

Abiotic stress conditions cause the activation of the phenylpropanoid biosynthetic pathway, generating an increase of different phenolic compounds to ameliorate plant performance under stress conditions. Nitrogen is an important plant macronutrient, and its availability determines the distribution of photosynthetic components between the primary and secondary metabolisms of the plant [77]. 

The effect of N limitation on phenolic accumulation is still little known, and the mechanisms are also controversial. The first could be the C/N ratio theory, where, under N deficiency, production and accumulation shift to carbon-based metabolites. Polyphenols, flavonoids and tannins do not contain nitrogen. The deamination theory is related to the phenylpropanoid pathway and the release of ammonia from phenylalanine, the precursor of phenolic compounds, by phenylalanine ammonia-lyase (PAL), which drives phenylpropanoid synthesis. Finally, the photoprotection theory is the capacity of phenolic compounds to reduce oxidative damage in plants when, in N deficiency conditions, increased ROS production occurs. In accordance with these theories, N limitation induces the plant to accumulate carbon-based assimilates by activating secondary metabolite biosynthesis.

Interestingly, many crop species have been reported to have an accumulation of phenolic content under N deficiency [78,79]. 

Reduced application of nitrogen in crop production is significant for ecological and health-related reasons; nitrogen limitation led to an increased content of polyphenols in leafy vegetables such as red and green leaf lettuce [78]. 

The application of vermicompost, with a lower content of total N and NO_3_^−^, could be in accordance with the mentioned theory. In a previous study on *Brassica campestris ssp. Chinensis*, the use of vermicompost significantly increased marketable yield, leaf nutrient content and antioxidant capacity. The beneficial effects could be due to the chemical compounds and physiological properties of vermicompost as well as to its effects on soil physical properties [80]. 

Under the treatments with vermicompost, biochars and corresponding mixtures, the contents of polyphenols, flavonoids and hydrolyzable tannins in Swiss chard leaves were found to be higher or similar to chemical fertilizer. The experimental treatments did not affect the content of condensed tannins. Biochars alone induced a slight reduction of condensed tannins; they protect the plants against pathogens and diseases and control the seeds’ permeability and dormancy; factors such as light could affect the biosynthesis of condensed tannins, as reported in *Lotus corniculatus* leaves [81]. In a greenhouse study on *Populus fremontii × angustifolia* hybrids, the condensed tannin content clustered separately from other metabolic profiles and seemed to be efficient for suppressing leaf expansion [82]. Swiss chard is a good source of phytochemicals; a previous study reported the concentration of phenolic acids in leaves, showing higher contents of syringic acid (45.1 mg/100 mg fw) and ferulic acid (10.8 mg/100 fw) and a minority amount of other phenolic acids (gallic, chlorogenic, vanillic, ferulic, *p-*coumaric acids; range 2–8 mg/100 fw) [6]. The most abundant flavonoid was kaempferol (5.8 mg/100 g fw), along with quercetin and myricetin; catechin, one of the monomers used to synthesize condensed tannins, is also found in Swiss chard leaves in small amounts (1.5 mg/100 g fw) [6]. The antioxidant activity is related to the polyphenolic compounds, including Swiss chard compounds; due to their chemical features, they are able to counteract and decrease the oxidative stress involved in several human diseases. Among the investigated specialized metabolite classes, a strong correlation was observed between hydrolyzable tannins and reducing power and DPPH-scavenging activity, with Pearson’s correlation coefficients, *r*, of 0.83 and 0.59, respectively. Tannins are considered antinutritional compounds, but recent evidence has shown that they can have health benefits, exhibiting marked antioxidant properties related to the prevention of cancer, osteoporosis and cardiovascular disorders as well as a preservative effect in food (food industry) [83]. Correlations were also observed between TPC and FRAP (*r* = 0.56) and DPPH (*r* = 0.34), as well as TFC and FRAP (*r* = 0.21).

## 4. Materials and Methods

### 4.1. Experimental Site, Materials Used in the Experiment and Analyses

The study was carried out at the University of Basilicata, located in Potenza (PZ, 40°38′ N–15°48′ E, 819 m a.s.l.), southern Italy, in the spring–summer season, from May to July 2021. 

The trial consisted of a pot experiment carried out in a greenhouse, under natural conditions of light and temperature, on Swiss chard (*Beta vulgaris* L. var. *cycla*) plants grown on soil treated with a chemical fertilizer, a compost and two biochar types. 

For the experiment setup, soil was collected in a farm located in southern Italy in the agricultural area of the Potenza district from the upper 0–30 cm soil layer. It was characterized by contents of sand, silt and clay, respectively equal to 66.1%, 11.5% and 22.4%, resulting in a sandy-loam texture, according to the classification of the USDA (United States Department of Agriculture). The soil was analyzed for the main physicochemical properties, such as pH and electrical conductivity (EC, mS m^−1^), total carbon (C, % dw), nitrogen (N, % dw) and organic carbon (Corg, % dw) contents, C/N ratio, and nitrate (NO_3_^−^, mg kg^−1^) and ammonium (NH_4_^+^, mg kg^−1^) contents, following the standard laboratory protocols reported in the Italian Official Gazette [84]. 

The chemical fertilizer was a commercial synthetic fertilizer, i.e., ammonium nitrate, with 34% total N (17% NO_3_^−^ and 17% NH_4_^+^). 

The compost was a commercial product, i.e., a vermicompost from cattle manure, purchased from a company in the Matera district (south Italy) that is specialized in the composting activity of different types of biomass to produce highly controlled organic amendments. The two biochar types were respectively obtained from wood chips and pruning vine (*Vitis vinifera* L.) biomasses. The wood chip biochar was purchased from a company in the Torino district (North Italy), specialized in the production of a commercial biochar by a pyrolysis plant of their own design, using wood chips and wood processing wastes from the cleaning of green areas and woods within a controlled supply chain. The vine pruning biochar was produced at the STAR*Facility Centre of Foggia University (south Italy) in a pilot-scale pyrolyzer plant equipped with a fixed-bed tubular reactor (30 L capacity), where pruning chips of about 50 mm, with 15% humidity, were treated at a temperature of 750 °C for 8 h and a heating rate of 10 °C min*^−^*^1^. 

The main physicochemical properties of vermicompost and biochars were determined before the trial started, according to the following analytical procedures. The pH was measured by a GLP 22+ pH meter (Crison Instruments, Barcelona, Spain) and the electrical conductivity (EC, mS m^−1^) by a GLP 31+ EC meter (Crison Instruments, Barcelona, Spain), using a dilution of 1:20 (*w*/*v*) vermicompost:deionized water or biochar:deionized water after 1 h of shaking and 5 min of equilibrium time. The determination of moisture, volatile solids, ash and fixed carbon contents (% dw) was according to the ASTM D7582 method using a thermogravimetric analyzer unit (LECO-TGA701). The determination of C, N, H and S contents (% dw) was according to the LECO-ASTM D5373 method, by dry combustion, using a CHNS elemental analyzer (CHNS LECO 680). Corg was determined, after destroying the carbonates with HCl, by combustion. The two biochars were also analyzed for oxygen (O, % dw) by calculating the following: O = 100-C-H-N-S-ash; carbon stability by determining the molar ratios H/Corg and O/Corg. The biochars had a C content within the EBC (European Biochar Certificate) threshold [85] and a Corg content according to the IBI (International Biochar Initiative) Class 1 standard [86]. The H/Corg molar ratio complied with both the EBC and the IBI Standard (H/Corg *≤* 0.7), and the O/Corg ratio that allowed us to differentiate biochar from other carbonization products [87] was according to the EBC and the IBI Standard (O/Corg *≤* 0.4).

Other details about the physicochemical properties determined in both soil and organic amendments are shown in Table 2.

### 4.2. Experimental Layout and Setup 

The experimental treatments consisted of: untreated soil or control (C); soil treated with ammonium nitrate (F) or vermicompost from cattle manure (V), both applied to provide 280 kg N ha^−1^; soil treated with biochar from wood chips (Bw) or biochar from vine prunings (Bv), both at 2% of the dry soil weight rate; soil treated with a mixture of biochar from wood chips and ammonium nitrate (Bw+F) or vermicompost (Bw+V) and a mixture of biochar from vine prunings and ammonium nitrate (Bv+F) or vermicompost (Bv+F), each of them applied as above. Particularly, based on the N content, the amount of vermicompost added to the soil corresponded to 1.5% of the dry soil weight rate. The 9 experimental treatments were arranged according to a completely randomized block design, with each of them replicated four times for a total of 36 experimental units. 

Before the trial started, both the soil and the two biochars were first air-dried, then crushed, and, finally, sieved at 2 mm. According to the experimental layout, the ammonium nitrate, vermicompost and biochars were added to the soil alone or in a mixture and, after a thoroughly mixing, square pots (13 cm length × 13 cm width × 24 cm height) were filled using 2 kg of experimental soil. Successively, soil water content was brought to water-holding capacity or field capacity (FC, % dry weight), previously determined for each experimental treatment at −0.03 MPa by using a pressure-plate apparatus (Soilmoisture Equipment Corp., Goleta, CA, USA). A single seedling was transplanted into the respective treatment pot and soil surface of each pot was covered to prevent losses of water by evaporation by using polythene beads placed to form a 3 cm layer. Plants were irrigated throughout the growing cycle every 2–3 days to compensate for transpiration losses, checked daily by weighing the pots at the same hour (9:00–10:00), by increasing soil water content to the FC of each experimental treatment at each watering.

Starting from two weeks after transplanting until the end of the growing cycle, five leaf harvests were carried out, on average, every ten days by picking all the mature leaves and fully expanded leaves, taking care not to damage the growing point of the newly formed basal leaflets, allowing for quick development.

### 4.3. Plant Measurements and Analyses

During the growth cycle, a set of plant measurements and analyses was carried out as follows. 

The leaf greenness or Soil and Plant Analysis Development (SPAD) index was estimated using a hand-held SPAD-502 m instrument (Konica-Minolta corporation, Ltd., Osaka, Japan). More specifically, before each leaf cut, the average SPAD index per plant was calculated from SPAD readings performed on all the fully expanded leaves ready to be harvested. Before each leaf cut, plant height (H), from the surface line to the top of the longest leaf, was also measured. After each leaf cut, leaf area (LA, cm^2^; using an LI-COR leaf area meter; Model 3100, Inc., Lincoln, NE, USA), fresh weight (fw, g) and dry weight (DW, g; using an analytical balance) were measured. In particular, DW was determined after drying the leaves in a ventilated oven at 65 °C. The leaf dry matter content (DM, % fresh weight) was then calculated.

At the end of the growth cycle, the yield parameters, such as the average plant height (H, cm), total leaf area (LA, expressed as m^2^ m^−2^) and average leaf dry matter content (DM, %), were obtained over the five leaf harvests, together with the total yield (yield, expressed as g fw m^−2^). 

Moreover, the water consumption (Wc, expressed as L m^−2^) over the entire growing cycle was determined by summing the amount of water supplied at each watering to each plant. Finally, the water-use efficiency (WUE, expressed as g fw L^−1^) was calculated as the ratio of yield to Wc.

The total nitrogen (total N) and nitrate (NO_3_^−^) contents were determined from the dried tissues of each leaf harvest by first cumulating and then grinding and homogeneously mixing them. Then, total N (% dw) was determined by the dry combustion of 2–5 g of plant material packed in tin foil capsules using an automated CHNS elemental analyzer (CHNS LECO 680). NO_3_^−^ was determined by extracting nitrates from 0.5 g of plant material with 50 ml 3.5 mmol l^−1^ Na_2_CO_3_ and 1.0 mmol l^−1^ Na_2_HCO_3_ using an ion exchange chromatograph (Dionex ICS-5000, Dionex Corporation, Sunnyvale, CA, USA) equipped with an IonPac AG14 precolumn and an IonPac AS14 separation column. The data were expressed as mg kg^−1^ fresh weight. 

### 4.4. Extraction Procedure of Leaf Tissues

For the determination of phytochemicals and antioxidant activity, one g of dried leaf sample was extracted three times with 20 mL of ethanol solvent at 37 °C (3 × 1 h). The supernatant was collected and filtered through Whatman paper no. 1, and the solvent was removed by a rotary evaporator under reduced pressure. The extraction yield was calculated (Table 3). 

### 4.5. Phytochemical Content Measurements

The total polyphenolic content (TPC) was determined by the Folin–Ciocalteu reagent method [88]. The TPC of the extracts was expressed as milligrams of gallic acid equivalents (GAE) per gram of fresh weight ± s.e.

The total flavonoid content (TFC) was determined using the aluminum chloride assay, as previously reported [89]. The absorbance was measured at 510 nm. Results were expressed as mg of quercetin equivalents (QE) per gram of fresh weight ± s.e.

The determination of condensed tannins was by vanillin assay [90]. Ethanol extracts (50 μL) were mixed with 200 μL of 1% vanillin–H_2_SO_4_ (70%) and allowed to react for 15 min at 35 °C in the dark (Abs, 500 nm). Results were expressed as mg catechin equivalent (CE) per g of fresh weight ± s.e.

The hydrolyzable tannins were evaluated by the KIO_3_ method [91]. Hydroalcoholic extracts (50 μL) and 200 μL of KIO_3_ aqueous solution (2.5% *w/v*) were heated at 30 °C (7 min). Tannic acid solution (0.039–5.00 mg/mL) was used as standard (550 nm), and results were expressed as mg tannic acid equivalent (TAE) per gram of fresh weight ± s.e.

### 4.6. Antioxidant Activity Measurement

Radical-scavenging ability was measured using the stable radical 2,2-diphenyl-1-picrylhydrazyl (DPPH) assay [92]. Absorbance at 515 nm was recorded. 

The ferric-reducing antioxidant power (FRAP) was based on the reduction of Fe^3+^ to Fe^2+^ by the action of electron-donating antioxidants [93]. Trolox was used as a standard for antioxidant activity. Results were expressed as milligrams of TE per gram of fresh weight ± s.e.

According to a previous study [93], the Relative Antioxidant Capacity Index (RACI) was calculated by the integration of the in vitro antioxidant data (DPPH and FRAP), including TPC. This can be considered an alternative assay to determine the total reducing capacity of the extracts. 

In the RACI, the standard scores were determined by the following equation:

(1) $$\frac{\left(x\;–\;\mu \right)}{\sigma }\;\;\;\;$$

where x is the raw data, μ is the mean, and σ is the SD. A high RACI score means high relative antioxidant activity.

### 4.7. Statistical Analysis

All the experimental data were preliminary checked for normality and homogeneity of variance and then analyzed by one-way ANOVA. When significant differences among the means were detected, they were separated by Tukey’s honest significance difference post hoc test at the 5% probability level. The JMP software package, version 15 (SAS Institute Inc., Cary, NC, USA), was used to carry out the statistical analysis.

## 5. Conclusions

The use of soil organic amendments, such as compost and biochar, deriving from the recycling of different types of organic residues and wastes, is nowadays reported as a win–win approach towards more environmentally friendly agriculture. In recent years, their application as partial or total substitutes for chemical fertilizers is of growing interest. 

The current study has evaluated the effects of soil treatment with a chemical fertilizer (ammonium nitrate), a vermicompost from cattle manure and two biochars from wood chips and vine prunings, respectively, on Swiss chard yield, phytochemical content and biological activity. 

Results showed a clear increase in plant height, leaf area and total yield following an application to the soil of vermicompost, both alone and in a mixture with biochar from vine prunings. Very interestingly, the enhancement of yield parameters and total yield were found to be similar to those observed in plants grown on soil treated with ammonium nitrate, both alone and in a mixture with biochar from vine prunings, suggesting that vermicompost is very beneficial to Swiss chard cultivation and has great potential as a substitute for chemical fertilizers in view of more sustainable cropping practices. 

Moreover, compared to ammonium nitrate, the use of vermicompost, both alone and in a mixture with the two biochars, respectively, also positively affected the nutritive and health quality of this specie. Indeed, these organic amendments clearly resulted in lower leaf total N content and NO_3_^−^ accumulation, with the latter below the safe limits established by the European Community for other leafy vegetables similar to Swiss chard (spinach, lettuce and rocket), as well as in increased phytochemical content and antioxidant activity. 

Despite the positive effect when mixed with vermicompost, both the two biochars applied alone did not influence either yield or leaf total N content, thereby showing very limited fertilizing value, which was also reflected in the undetectable leaf NO_3_^−^ content. However, considering that biochar nutrient availability is highly variable, depending on the feedstock types used and pyrolysis conditions applied, further pot and field-scale experiments should be carried out to better understand the fertilizing effect of this organic amendment. Such an investigation becomes even more relevant in light of the possibility of using biochar in organic farming throughout Europe as a fertilizer/soil conditioner listed in Annex I of EC Regulation No. 889/2008. The implementing Regulation No 2019/2164 has been in force since 2020.

The conclusions that can be drawn from the findings of the present study indicate that organic amendments, particularly vermicompost, can be used in Swiss chard cultivation as a suitable alternative to chemical fertilizers to meet the targets of yield and health quality of vegetable products, also contributing to the recycling of organic wastes and the development of sustainable agriculture. 

## Figures and Tables

**Table 1:** Plant height (H, cm), leaf area (LA, m^2^ m^−2^), leaf dry matter (DM, %), total yield (Yield, g fw m^−2^), water consumption (Wc, L m^−2^) and water-use efficiency (WUE, g fw L^−1^) of Swiss chard.

Treatment	H (cm)	LA (m^2^ m^−2^)	DM (%)	Yield (g fw m^−2^)	Wc (L m^−2^)	WUE (g fw L^−1^)
C	9.7 ± 0.3 c	2.4 ± 0.1 b	7.7 ± 0.6	1066 ± 107.3 b	151 ± 0.6 ab	7.1 ± 0.7 c
F	15.1 ± 1.4 a	5.5 ± 0.9 a	7.9 ± 0.3	2798 ± 38.7 a	163 ± 8.2 ab	16.9 ± 1.9 a
V	13.6 ± 0.4 ab	5.2 ± 0.1 a	6.9 ± 0.4	2618 ± 35.3 a	159 ± 4.2 ab	16.6 ± 0.5 a
Bw	9.8 ± 0.3 c	2.4 ± 0.0 b	7.8 ± 0.3	1056 ± 39.5 b	140 ± 1.5 b	7.5 ± 0.3 bc
Bv	10.6 ± 0.3 bc	2.6 ± 0.1 b	7.2 ± 0.3	1184 ± 65.7 b	139 ± 5.2 b	8.6 ± 0.7 bc
Bw + F	13.2 ± 1.1 ac	4.3 ± 0.6 ab	8.0 ± 0.3	2070 ± 337.1 ab	163 ± 6.9 ab	12.5 ± 1.7 ab
Bw + V	13.2 ± 1.4 ac	3.8 ± 0.6 ab	8.1 ± 0.4	1898 ± 372.4 ab	150 ± 9.1 ab	12.4 ± 1.7 ac
Bv + F	14.3 ± 0.5 a	5.4 ± 0.6 a	7.1 ± 0.2	2676 ± 369.5 a	173 ± 9.3 a	15.3 ± 1.4 a
Bv + V	14.4 ± 0.4 a	4.5 ± 0.0 a	7.1 ± 0.3	2461 ± 32.8 a	165 ± 1.6 ab	14.9 ± 0.3 a
*Significance*	***	***	ns	***	**	***

C, control; F, ammonium nitrate; V, vermicompost; Bw, biochar from wood chips; Bv, biochar from vine prunings; Bw+F and Bv+F, biochars respectively mixed with ammonium nitrate; Bw+V and Bv+V, biochars respectively mixed with vermicompost. Values are means (n = 4) ± s.e. In each column, means followed by different letters are significantly different (*p* ≤ 0.05; Tukey’s test). **, F test significant at *p* ≤ 0.01; ***, F test significant at *p* ≤ 0.001; ns, not significant.

**Table 2:** Main physicochemical properties of the soil, vermicompost and biochars used in the experiment.

Property	Soil	Vermicompost	Wood Chip Biochar	Vine Pruning Biochar
pH (-)	7.6 ± 0.1	7.6 ± 0.1	8.9 ± 0.1	10.6 ± 0.1
EC ( mS m^−1^)	0.6 ± 0.1	265.0 ± 0.0	52.0 ± 0.0	249.0 ± 0.0
Moisture (% dw)	6.0 ± 0.1	4.0 ± 0.2	5.6 ± 0.1	15.3 ± 0.3
Volatile solids (% dw)	-	27.5 ± 0.6	42.3 ± 0.4	15.3 ± 0.3
Ash (% dw)	-	72.2 ± 0.6	4.4 ± 0.2	9.9 ± 0.0
Fixed carbon (% dw)	-	0.2 ± 0.0	53.3 ± 0.2	74.8 ± 0.3
C (% dw)	0.8 ± 0.0	11.3 ± 0.0	68.3 ± 0.1	67.7 ± 0.9
H (% dw)	-	1.5 ± 0.1	4.0 ± 0.0	2.1 ± 0.0
N (% dw)	0.2 ± 0.0	1.5 ± 0.0	1.0 ± 0.0	1.0 ± 0.0
S (% dw)	-	0.3 ± 0.0	0.03 ± 0.0	0.2 ± 0.0
C_org_ (% dw)	0.6 ± 0.0	7.8 ± 0.1	66.3 ± 0.1	67.0 ± 0.9
O (% dw)	-	5.2 ± 0.2	22.3 ± 0.3	17.9 ± 1.5
H/C_org_ (-)	-	-	0.7 ± 0.0	0.4 ± 0.0
O/C_org_ (-)	-	-	0.4 ± 0.0	0.2 ± 0.0
C/N (-)	3.9 ± 0.1	5.0 ± 0.2	67.2 ± 2.0	66.2 ± 0.1
NO_3_^−^ (mg kg^−1^)	49.1 ± 1.9	11574 ± 445	<0.1	<0.1
NH_4_^+^ (mg kg^−1^)	<0.1	27.7 ± 0.8	<0.1	<0.1

Values are means (n = 3) ± s.e.

**Table 3:** Extraction yield of hydroalcoholic extracts from Swiss chard leaves.

Treatment	Extraction Yield (%)
C	5.10
F	5.79
V	5.77
Bw	5.91
Bv	5.69
Bw + F	6.67
Bw + V	6.11
Bv + F	5.51
Bv + V	5.77

C, control; F, ammonium nitrate; V, vermicompost; Bw, biochar from wood chips; Bv, biochar from vine prunings; Bw+F and Bv+F, biochars mixed with ammonium nitrate; Bw+V and Bv+V, biochars mixed with vermicompost.

## Data Availability

The data presented in this study are available upon request from the corresponding author.
